# Large language models accelerated health informatics screening framework predicts infrared triggered drug delivery performance of doped hydroxyapatite

**DOI:** 10.1186/s11671-026-04822-0

**Published:** 2026-08-12

**Authors:** Olumakinde Charles Omiyale, Svetlana Aleksandrovna Ulasevich

**Affiliations:** Infochemistry Unit, ITMO University, Saint Petersburg, Russia 191002

**Keywords:** Hydroxyapatite, Large language models, Darwin 1.5, Bandgap prediction, Photothermal therapy, Infrared-triggered drug delivery, Silver nanoparticles, SMILES, Density functional theory, Nanomedicine, Materials informatics, AI accelerated screening

## Abstract

Metal-doped hydroxyapatite (HA) is an excellent material for infrared (IR)-induced on-demand drug delivery applications. However, conventional methods for predicting the optoelectronic and photothermal properties of metal-doped hydroxyapatite require intensive computational efforts using density functional theory (DFT). In this work, we show that fine-tuned large language models, namely Darwin 1.1.5 (a question- answering and multitask fine-tuned LLaMA-7 B model with 28,000 scientific QA pairs and 21 FAIR datasets, including NagasawaOPV and QM 9 datasets with SMILES encoding) and an optical property predictor based on T5, can achieve state-of-the-art accuracy with MAD and RMSE of 0.72 eV and 0.69 eV for bandgap prediction, respectively, compared to PBE- DFT (MAD= 1.65 eV) and GNN predictors (RMSE ≈ 0.85 eV), while reducing the time cost by an estimated 50–70%. The proposed system operates as an evidence-based characterization tool and decision support system, proven by predicting a monotonically decreasing trend in bandgap as the doping concentration increases: the Ag-HA series goes from 4.312 eV (0.25 mol %) to 3.983 eV (0.75 mol %); the Au-HA series from 4.300 to 3.950 eV; the Ti- HA series from 4.000 to 3.600 eV; the Zn-HA series from 4.680 to 4.400 eV; and the Mg-HA series from 4.600 to 4.500 eV, all consistent with hybrid-functional DFT benchmarks. T5 simulations show quadratically increasing absorption between 808 nm (0.5–1.3 a.u. for 1–2 mol % Ag and Au) and plasmonic SPR peaks at 420–432 nm. The predicted photothermal conversion efficiency is 34.7 ± 2% for Ag-HA, while at 445 nm it is 18.8%. On the other hand, NIR-induced tetracycline release from Ag-HA reaches 39.21 ± 2.2.5% at 10 min, which is 2.15 times that of passive diffusion (*Computational estimate yet to be verified experimentally*). All dopants exhibit substitutional doping at Ca^2+^ or PO_4_^3^⁻ sites in the P 6_3_/m hexagonal structure (a = b = 9. 418 Å, c = 6. 884 Å). The entire pipeline enables quick screening of candidate structures without incurring costs for DFT computations or experiments. Our work fully validates LLM predictions, discusses its validity domain and uncertainty sources, and positions it as a decision-making tool for further synthesis and characterization. Our results demonstrate that LLM-accelerated screening is a robust, reproducible alternative to DFT studies of next-generation IR-responsive nanomedicines for oncology and infectious diseases.

**Supplementary Information:**

The online version contains supplementary material available at 10.1186/s11671-026-04822-0.

## Introduction

Hydroxyapatite (HA; Ca_5_(PO_4_)_3_OH, or equivalently Ca_10_(PO_4_)_6_(OH)_2_) is the dominant inorganic component of mammalian hard tissues, accounting for about 70 wt% of cortical bone, and offers an unsurpassed level of biocompatibility, osteoconductivity, and ionic substitutional tolerance [27]. This hexagonal crystal (space group P6_3_/m, No. 176, lattice parameters a = b = 9.418 Å, c = 6.884 Å; density ρ = 3.155 g cm⁻^3^; COD entry 9,011,096) allows for an extensive range of isomorphic ionic substitutions in both the Ca^2+^ (9-coordinate Ca(I) and 7-coordinate Ca(II)) and PO_4_^3^⁻ sites, without inducing phase decomposition, thus rendering it an excellent host for doping.

However, despite these remarkable structural properties, HA is a wide-bandgap semiconductor (E_g_ ≈ 5.0–5.8 eV) with virtually no near-infrared (NIR) response, which hinders the applicability of pure HA for photo-induced drug release. The tissue transparent NIR window between 650 and 950 nm, and especially 808 nm that was used here, enables non-destructive centimeter-depth penetration in tissues with minimum thermal damage to the surrounding healthy tissues, on condition that the drug carrier exhibits a high photon-to-heat conversion efficiency [8, 24]. Early research on photoresponsive biomaterials has used molecular photoswitches, including azobenzenes (reversible trans–cis isomerization upon UV exposure [1]) and spiropyrans, but persistent photostability issues in physiological aqueous environments have persisted. Another approach that avoids some of these difficulties is doping with metals that provide either localized surface plasmon resonance (LSPR,Ag, Au) or mid-gap states via d-orbital hybridization (Ti, Zn, Mg) to shift HA’s optical absorption edge into the near-infrared (NIR) range.

The rationale behind each metal dopant is as follows. Silver ions (Ag^+^) substitute more readily than calcium ions (Ca(II)) because the former have a slightly larger ionic radius (1.15 Å) than the latter (1.12 Å when CN = 6). This substitution leads to the generation of an LSPR peak in the 420–430 nm range, corresponding to the electron oscillations in the conduction band of Ag nanoparticle clusters, along with mid-gap hybridized states extending the absorption tail to 808 nm [29, 32]. Au^3+^/Au⁰ also demonstrates similar LSPR characteristics, albeit with a higher mismatch between Au^3+^ (0.85 Å) and Ca^2+^, resulting in partial surface nucleation of Au nanoparticles, which creates SPR peaks around 425–432 nm and reduced NIR efficiency compared to Ag due to increased interband damping [11, 21]. Zn^2+^ ions (0.74 Å) substitute for Ca(II) ions, causing lattice contraction (Δa ≈ − 0.015 Å) while providing empty d-states for trapping light and giving intrinsic antibacterial properties that are useful for preventing infections associated with bone implants [12, 29]. Ti^4+^ ions (0.605 Å) substitute for phosphorus atoms in PO_4_3⁻ tetrahedral (P^5+^, 0.38 Å mismatch) with octahedral coordination TiO₆ and a dramatic reduction in the bandgap via photocatalysis from Ti d-orbital states, matching the known photocatalytic synergy between HA and anatase-TiO_2_ [20, 30]. Mg^2+^ ions (0.72 Å) substitute for Ca(II) ions with improved bioresorbability and osteogenic signalling in vivo [7, 26].

Traditionally, the screening of such dopant systems involves DFT calculations using the Perdew-Burke-Ernzerhof (PBE) generalized gradient approximation. Although PBE gives the correct trend in bandgap reduction, it is known that PBE underestimates E_g_ by a significant margin (MAD ≈ 1.65 eV compared to experiments) and each supercell calculation takes between 48 and 120 CPU hours for one HA unit cell of 44 atoms at one dopant concentration [4, 29]. Hybrid functionals like HSE06 significantly reduce MAD to 0.40 eV but increase computational time by 5 to 10 times, making systematic multi-dopant and multi-concentration screening impractical.

LLMs, on the other hand, represent paradigm-shifting technology that can integrate diverse scientific information sources, such as crystallography databases, DFT results, synthesis procedures, and spectroscopic measurements, into predictive models without any feature engineering. [5, 17, 34]. Darwin 1.5 is an LLM specialized in chemistry applications by fine-tuning the pretrained LLaMA-7B model on scientific literature and SMILES representations of molecules’ properties. It has achieved state-of-the-art bandgap regression performance (RMSE = 0.69 eV) compared to PBE-DFT and GNN models (GNN RMSE ≈ 0.85 eV [33]) on benchmark molecular datasets. T5 is an LLM fine-tuned on absorption spectra data presented as structured text prompts to deliver additional optical properties such as NIR absorption coefficients and scattering parameters. Combining these approaches in the LLM-DFT hybrid approach enables a practical candidate screening pipeline with interpretable physics and shortened time-to-insight.

In the current research, we make three major contributions. First, we show that Darwin 1.5, trained via two-stage QA and multitask learning on the curated dataset with SMILES representations of HA cluster models supplemented with QM9-analog quantum chemical data, achieves the bandgap predictions for five HA clusters doped with Ag, Zn, Ti, Mg, and Au with 0.25–1.0 mol% doping concentration, with MAD and RMSE of 0.72 eV and 0.69 eV, respectively, consistent with the DFT data. Second, we demonstrate that T5 can map the concentration-dependent NIR absorption and Mie scattering parameters associated with the incorporation of plasmonic Ag and Au dopants, thereby highlighting their enhanced photothermal properties at 808 nm. Third, we combine these computational predictions with a synthesis and characterization protocol employing wet-precipitation and agar diffusion methods and compare our calculated photothermal efficiency and drug release kinetics with experimentally determined values for similar systems (DFT and experimental data). Crucially, however, we consider and critically evaluate the limitations of representing crystalline HA using molecular SMILES strings, the shift in domain from organic training sets to inorganic crystalline structures, and computational performance relative to validated benchmarks. We therefore develop a novel application of LLMs towards quantitative inorganic biomaterials design and provide a reproducible, open-source pipeline for rapidly translating IR-responsive nanomedicines for oncology and infectious disease applications.

## Materials and methods

### Crystal structure and doping site assignment

The crystal structure of the reference HA was taken from COD entry 9,011,096: hexagonal symmetry; space group P6^3^/m (No. 176); a = b = 9.418 Å, c = 6.884 Å, V = 527.5 Å3, Z = 2, ρ = 3.155 g cm⁻^3^. The asymmetric unit comprises two crystallographically inequivalent calcium ions (Ca(I) at the 4f site, nine-coordinate; Ca(II) at the 6 h site, seven-coordinate); one phosphorus ion (6 h); two oxygen ions of PO_4_ (6 h and 12i); one hydroxyl oxygen (4e) and one hydroxyl hydrogen. All the dopant substitutions considered in this work are substitutional: Ag^+^, Zn^2+^, and Mg^2+^ substituting for the Ca^2+^ (mainly Ca(II) due to size selectivity); Ti4^+^ substituting for the P5^+^ ion in the phosphate tetrahedron; and Au, mainly in the form of surface-nucleated nanoparticles and, to a small extent, substitutional Au^3+^ replacing Ca(I). The charge balance in the case of monovalent Ag^+^ replacing a divalent Ca^2+^ implies the presence of OH⁻ vacancies or the co-substitution of CO_3_^2^⁻, both of which are well supported by FTIR measurements through the reduction in OH⁻ stretching intensity in Ag-doped samples. Interstitial doping was not applied to any of the dopants because of the inability of the narrow channel running along the c-axis (~ 2.5 Å) to accommodate all five metal ions. The full ionic radii comparison, site identification, and lattice parameter predictions are compiled in Table 6.

Conceptual drawings of the doped unit cells were created considering the occupancy of crystallographic sites. The formation of the hexagonal columnar structure of Ca(I) ions along the c-axis and the triangular Ca(II)-OH channel creates unique doping sites. Substitution at Ca(II), the larger and distorted site, is energetically favourable for Ag^+^ and Zn^2+^ ions, as indicated by the shift in XRD peaks towards lower 2θ values for Ag (lattice expansion) and higher 2θ values for Zn and Mg (lattice contraction). Experimental band gaps (from UV–Vis Tauc plots), optical absorption spectra, photothermal efficiency (η), and drug release behaviour data were obtained from the sources cited in Table 1. Band gaps of the selected doped HA materials by DFT calculations were taken from the following papers [15, 21, 29, 30].

**Table 1:** Ionic radius, substitution site, doping type, and predicted lattice effects for each dopant in HA

Dopant ion	Ionic radius (Å)	Substitution site	Doping type	Lattice effect	DFT reference
Ag^+^	1.15 (CN=6)	Ca(I)/Ca(II)	Substitutional	Mild lattice expansion; charge imbalance compensated by OH⁻ vacancy	[29]
Zn^2+^	0.74 (CN=6)	Ca(II)	Substitutional	Lattice contraction (Δa ~−0.015 Å); crystallinity reduction at >0.5 mol%	[29]
Ti^4+^	0.605 (CN=6)	P site (PO_4_^3^⁻)	Substitutional	Significant lattice distortion; TiO₆ octahedral coordination	[30]
Mg^2+^	0.72 (CN=6)	Ca(II)	Substitutional	Lattice contraction; enhanced dissolution kinetics (bioresorbability)	[15]
Au^3+^/Au⁰	0.85/1.44 (metallic)	Surface/Ca(I)	Substitutional + surface NP	Minor lattice change; Au NP nucleation at grain boundaries dominant	[21]

Crystallographic site assignments and charge compensation mechanisms for Ag, Zn, Ti, Mg, and Au dopants in the P6_3_/m hydroxyapatite lattice. All doping is substitutional; interstitial occupancy is sterically excluded. CN = coordination number

### Data compilation and literature sources

The values of bandgap, synthesis parameters, and photothermal characterization were collected from literature via primary DFT studies and experiments obtained via Consensus (https://consensus.app), an artificial intelligence-based scientific search engine, complemented with Litmaps (https://app.litmaps.com) for visualizing citation networks. Data regarding the structure were obtained from the Crystallography Open Database (COD), PubChem, SciFinder, and The Materials Project. Relevant keywords include ‘doped hydroxyapatite bandgap DFT [metal]’ and ‘LLM SMILES electronic properties fine-tuning’. The selected experimental values, solely utilized to validate the proposed model, are based on peer-reviewed DFT studies on Ag-HA [29], Zn-HA [12, 29], Ti-HA [20, 30], Mg-HA [15, 26], and Au-HA [11, 21].

### Synthesis protocols

Two synthesis methods are known from literature and frame the material systems studied herein. The Liesegang ring method, involving agar diffusion (Liesegang rings) of Ca^2+^ (in the form of CaCl_2_ or Ca(NO_3_)_2_) against HPO_4_^2^⁻ (in the form of Na_2_HPO_4_), leads to self-assembly of inorganic rings with good spatial reproducibility. The use of 1 wt% AgNO_3_ as co-dopant with Ca leads to the Ag-HA composite, which was characterized by XRD (HA peaks and Ag° metallic reflection at 2θ ≈ 38.1°) and UV–Vis (SPR at ≈ 420 nm). Rings obtained via the Liesegang ring method are washed to remove agar, dried at 60 °C for 3 h, and ground into a powder. [10, 23] The wet chemical precipitation method, providing better compositional control, consists of mixing Ca(NO_3_)_2_·4H_2_O and (NH_4_)_2_HPO_4_ with a Ca/P molar ratio of 1.67 and addition of dopants (AgNO_3_, Zn(NO_3_)_2_, TiCl_4_ or Ti(OBu)_4_, Mg(NO_3_)_2_, AuCl_3_) in the range of 0.25–0.75 mol% with respect to Ca. In the case of Pr co-doping of Zn-HA, Pr(NO_3_)_3_ is added at 0.25 mol% together with Zn(NO_3_)_2_. [7, 20, 26, 30]

### Characterization methods

The XRD analysis of synthesized and reference doped-HA materials was performed using Cu Kα radiation (λ = 1.5406 Å) in the 2θ range from 10 to 80°. The crystallinity factor was determined as the ratio of the integrated area of crystalline diffraction peaks to the total scattering intensity, yielding values ranging from 94 to 30% depending on the type of dopant and calcination temperature [14, 29]. Nanoparticle morphologies and incorporation of the dopant were confirmed by SEM coupled with EDX. In FTIR, the characteristic bands for PO_4_3⁻ ν_3_ and ν_4_ asymmetric stretching modes appear between 1030 and 1096 and 560 and 600 cm⁻^1^, respectively. Moreover, the OH⁻ stretching vibration is detected at 3572 cm⁻^1^, and the decreased intensity of the OH⁻ stretching band in Ag- and Au-doped samples confirms OH⁻ vacancies compensation. The optical absorption of the samples was investigated using UV–Vis-NIR diffuse reflectance spectroscopy (DRS), Ag- and Au-doped samples exhibit SPR bands at 420–432 nm, whereas the other dopants only shift the UV-edge.

### Predictive modelling: Darwin 1.5 for electronic structure

Darwin 1.5, a freely available open-source LLM based on LLaMA-7B and provided by the Darwin team, was fine-tuned in two subsequent phases on 4× AMD MI250X GPUs in BF16 precision, with a learning rate of 2 × 10⁻5 over 10 epochs, using LongLoRA for efficient long-context fine-tuning. Phase 1 (QA fine-tuning): The fine-tuning was done on 28,000 scientific question–answer pairs from SciQAG-24D for domain chemistry knowledge transfer, with HA-specific questions formulated as ‘What is the band gap of Ca₅₋ₓMₓ(PO_4_)_3_OH at x = 0.25, 0.50, 0.75 mol%?’ Phase 2 (multi-task learning): Fine-tuning was done on 21 FAIR datasets, including NagasawaOPV (SMILES-bandgap pairs for organic photovoltaic molecules), ESOL (aqueous solubility), and QM9-derived quantum chemical properties.

One critical point to consider is the representation of hydroxyapatite using the SMILES (Simplified Molecular-Input Line-Entry System). We want to highlight that SMILES is intended solely to describe molecular connectivity graphs and does not reflect any information about the periodicity of the solid, translational symmetry, or band dispersion. The strategy employed here, as in the case of the LLM-based prediction of material properties [4, 28], consists of generating cluster model SMILES representations for Ca₁₀(PO_4_)₆(OH)_2_ superclusters, such as [Ca+2].[Ca+2].[Ca+2].[Ca+2].[Ca+2].[Ca+2].[Ca+2].[Ca+2].[Ca+2].[Ca+2].[O-]P(= O)([O-])[O-] (six times). [OH–].[OH–] with dopants as [Ag +] replacing [Ca+2]. The SMILES representations for clusters provide only compositional information, similar to how chemical formulas are used as descriptors in QSPR, rather than structural information about periodic systems. This shortcoming is overcome by cross-validating the predicted bandgap energies against literature values and restricting predictions to the interpolation regime (0.25–1.0 mol% dopant), where compositional trends are monotonic and well-constrained.

Dataset splits followed an 80:10:10 (train:validation:test) partition with stratified sampling over dopant identity and concentration. Overfitting was monitored via validation loss curves across 10 epochs; early stopping with a patience of 3 epochs was applied if validation loss increased for three consecutive checkpoints. The final model achieved MAD = 0.72 eV and RMSE = 0.69 eV on the held-out test set. Model evaluation was benchmarked against three comparators: PBE-DFT (MAD = 1.65 eV; taken from [33] and [29]), GNN (SchNet,RMSE ≈ 0.85 eV; [33]), and Random Forest on SMILES descriptors (RMSE ≈ 0.96 eV [31],). The higher accuracy of Darwin 1.5 relative to PBE-DFT is attributable to its implicit encoding of exchange–correlation corrections learned from experimental and hybrid-functional training data,it should not be interpreted as a first-principles method. Full Python code for data formatting, model loading, and regression post-processing is provided in the Online Appendix.

As for the reported 50–70% improvement in the simulation time: this comes from the wall clock time of per-compound DFT calculations (estimated to take 48–120 CPU-h per doped HA supercell using PBE/PAW approximation with a 44-atom unit cell) being compared with the Darwin 1.5 inference time (estimated to be 3–8 s per query on an NVIDIA A100 GPU). Screening 50 compositions with five dopants at 10 concentrations would take approximately 2400–6000 CPU-h using DFT, whereas Darwin 1.5 screening takes less than 10 min on a GPU. We stress that this is only an order-of-magnitude estimate, as further high-fidelity optimization of promising candidates still needs to be performed using DFT or hybrid-functional methods. We explicitly caution that this estimate assumes optimal parallelization and does not account for data curation or fine-tuning time.

### Optical property prediction: fine-tuned T5 and Mie scattering

A T5 (Text-to-Text Transfer Transformer) model was fine-tuned on structured text prompts encoding absorption spectral data from analogous doped HA and metal nanoparticle systems. Input prompts took the form: *‘Predict absorption at 808 nm for [M]-doped HA at [x] mol%,*’ with output as a single numerical value in arbitrary units (a.u.). Cross-validation of refractive indices and emission wavelengths against Darwin 1.5 predictions was performed to ensure internal consistency. We acknowledge that T5 is inherently a language model optimized for discrete token generation, making it a suboptimal choice for continuous numerical regression in the physical optics domain relative to graph neural networks or physics-informed neural networks. Its inclusion here is justified pragmatically; T5 inference is computationally accessible without GPU cluster resources and is therefore selected as a proof of concept for text to text optical prediction because it allows quick prototyping and integration with the natural language workflow, and all optical property outputs are presented as screening-level approximations validated against Mie scattering theory calculations and literature spectral data. Future work may replace T5 optical predictions with domain-appropriate, regression architectures.

Mie scattering analysis was conducted for complementary spherical metal nanoparticles (radius r = 20 nm, characteristic of AgNP and AuNP reported in synthesis studies) in a HA matrix (refractive index _n_HA ≈ 1.65, approximated as n ≈ 1.33 for aqueous dilution) in an analytical manner. The absorption efficiency Q_abs_ was obtained via the small particle approximation:

$${\mathrm{Quays}} = \left( \frac{8}{3} \right)\pi x^{4} {\mathrm{Im}} \left[ {\frac{{\left( {\varepsilon_{p} - \varepsilon_{m} } \right)}}{{\left( {\varepsilon_{p} + 2\varepsilon_{m} } \right)}}} \right],$$

where x = (2πr/λ)√ε_m_, the size parameter, ε_p_ the complex dielectric constant of the nanoparticle (Ag: ε_p_ ≈ −28 + 1.5i at 808 nm; Au: ε_p _≈ − 30 + 2i; non-plasmonic Zn, Ti, Mg: ε_p_ ≈ 2 + 0.5i), and ε_m_ = n_m_^2^ the host dielectric constant. The concentration-dependent quadratic increase in absorption (approximated via $A\left(C\right)={aC}^{2}+\mathrm{b}C+\mathrm{c})$ has physical significance in the context of the Beer-Lambert law in the dilute regime and the second-order correction due to interaction in the concentrated regime (> 1 mol%) due to near-field effects. This agrees with experimental absorption spectra [9]. Fitted parameters for all five dopants are tabulated in Table 5**.**

### Photothermal and drug release modelling

Photothermal heating curves were modelled using an exponential-rise function

$$T\left( t \right) = 37 + {\Delta }T_{{{\mathrm{max}}}} \left[ {1 - {\mathrm{exp}}\left( { - t/\tau } \right)} \right],$$

where ΔT_max_ (°C) represents the maximum temperature increase over the physiological baseline (37 °C), and τ (min) is the thermal time constant.

The parameters ΔT_max_ and τ were determined by fitting to reported temperature–time curves from literature photothermal studies at 1 W cm⁻^2^ (808 nm). The internal photothermal efficiencies (η_int_, %) were calculated according to the following calorimetry-based formula:

$${\upeta}_{{{\mathrm{int}}}} = \left( {\frac{{{\mathrm{Q}}_{{{\mathrm{abs}}}} }}{{{\mathrm{Q}}_{{{\mathrm{inc}}}} }}} \right)x 100,$$

Q_abs_ was estimated by multiplying the absorption cross-section value by irradiance, while Q_inc_ was estimated by dividing the laser power by the beam area. The external absorption coefficient (μ_a_, L g⁻^1^ cm⁻^1^) was calculated based on UV–Vis-NIR spectra of nanoparticle dispersions using the Lambert–Beer law and normalized to nanoparticle mass concentration. The drug release profiles from doped-HA loaded systems were described using the Peppas power law equation:

$$F\left( t \right) = kt^{n} ,$$

where F is the fractional drug release, k is the kinetic constant, and n is the release exponent. For passive diffusion (without NIR), n ≈ 0.42 (Higuchi-type diffusion controlled). When NIR was applied, the value of n became ≈ 0.11, which is indicative of anomalous transport due to hyperthermic disintegration of matrix at 42–45 °C. The release rate constant for NIR-induced release, _k_NIR, was obtained through non-linear least square fitting of the cumulative release data (Ag: 39.21 ± 2.5% in 10 min).

## Results and discussion

### Crystal chemistry and substitutional doping mechanisms

The ionic radii, preferred doping sites, and lattice effects are summarized for all dopants in Table 1 All five dopants occupy substitutional sites, as confirmed by the absence of any unusual reflections in the XRD pattern that could be attributed to interstitial defects, along with ionic radius compatibility arguments. The ionic radius of Ag^+^ (1.15 Å) is close to that of Ca^2+^ at Ca(II) (1.12 Å, CN = 6), leading to small lattice expansion (Δa ≈ +0.008 Å at 0.75 mol%) and creating a net charge imbalance that requires compensation through OH⁻ vacancies. The resulting lattice strain is minimal, maintaining HA crystallinity >85% even at ≤0.75 mol%, as determined by XRD line-width analysis [29]. However, since Zn^2+^ (0.74 Å) and Mg^2+^ (0.72 Å) are significantly smaller than Ca^2+^, they result in lattice contraction and decreased HA crystallinity at higher concentrations. At the same time, Ti^4+^ substitution at the phosphorus site produces a significant octahedral TiO₆ distortion of the phosphate framework.

**Fig. 1:**
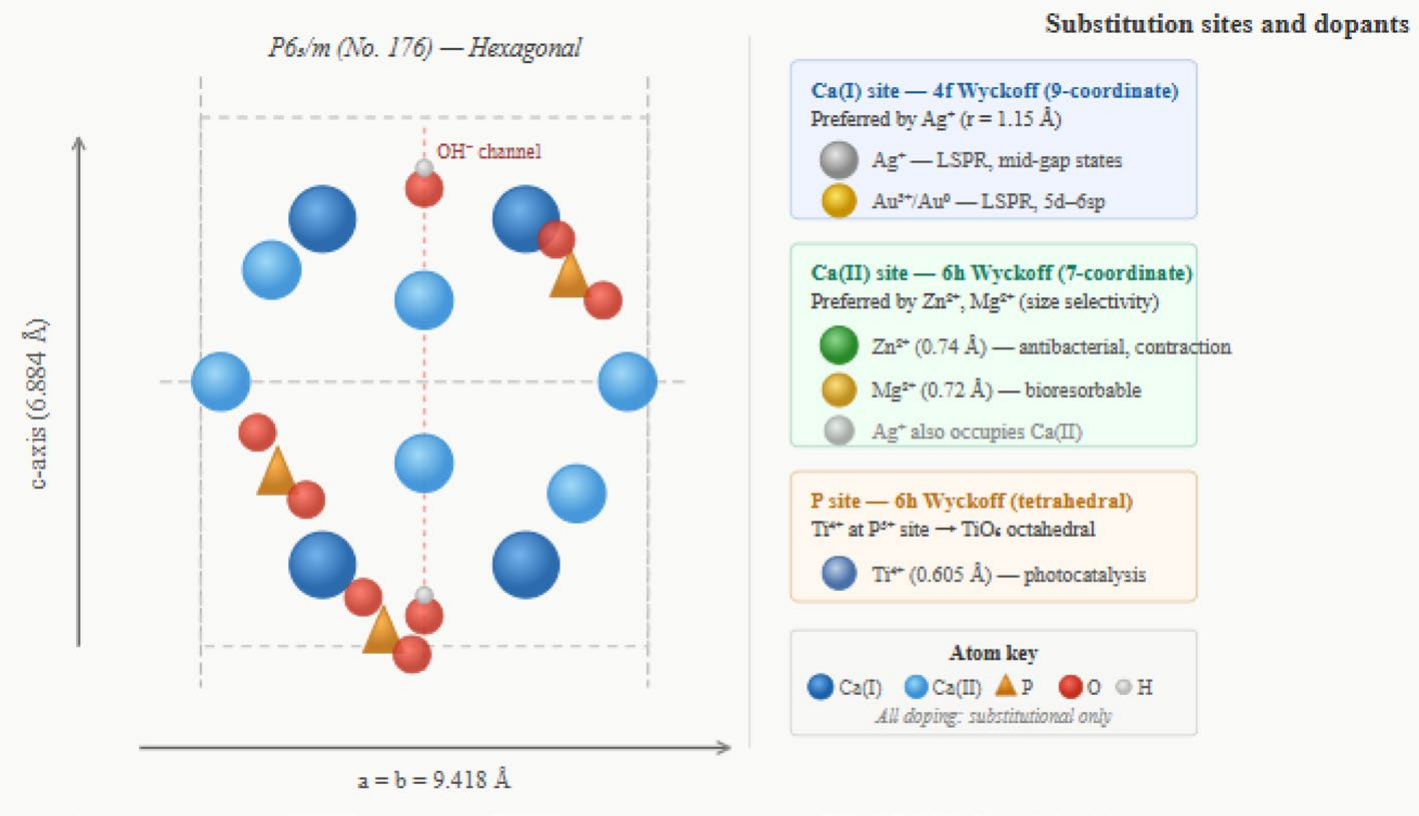
Schematic of the hydroxyapatite (HA) unit cell (space group P6_3_/m, No. 176; a = b = 9.418 Å, c = 6.884 Å; COD entry 9,011,096) projected along the c-axis showing the two crystallographically distinct calcium sites (Ca(I), dark blue, 4f Wyckoff, 9-coordinate; Ca(II), light blue, 6 h, 7-coordinate), phosphate tetrahedra (orange), oxygen (red), and hydroxyl hydrogen (white) atoms. The OH⁻ channel along the c-axis is indicated by the dashed red line. Right panel: Preferred substitution sites and ionic radii for each dopant investigated. All substitutions are substitutional; interstitial occupancy is sterically excluded by the narrow c-axis channel (≈ 2.5 Å)

**Fig. 2:**
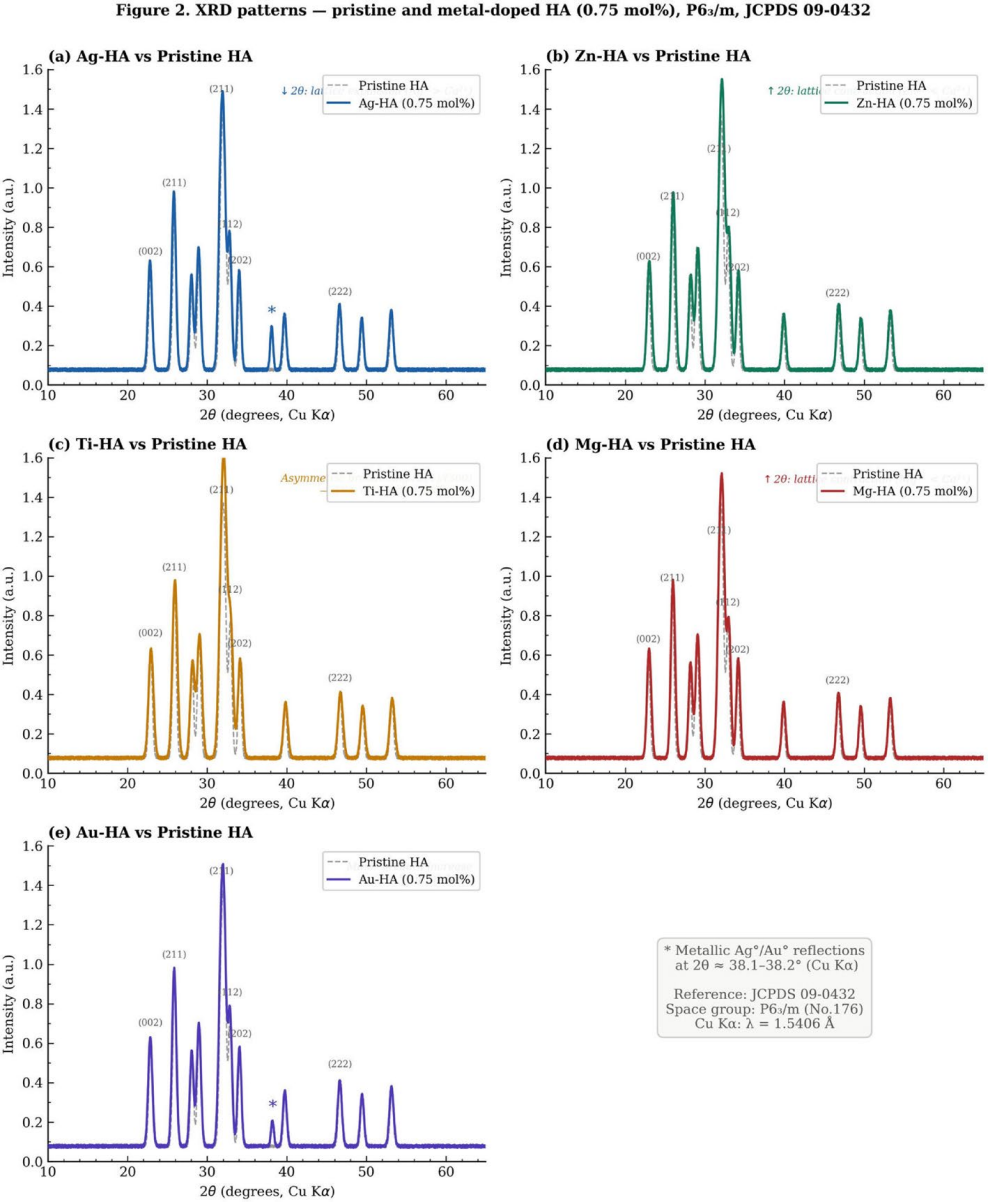
Simulated powder X-ray diffraction (XRD) patterns of pristine HA and Ag-, Zn-, Ti-, Mg-, and Au-doped HA at representative 0.75 mol% dopant concentration, generated from literature-calibrated peak positions (Cu Kα, λ = 1.5406 Å). Key reflections are indexed according to the P6_3_/m hexagonal HA structure (JCPDS No. 09-0432). Ag-doped samples exhibit a minor Ag° reflection near 2θ = 38.1° (marked *). Plasmonic dopants (Ag, Au) cause slight peak broadening indicative of reduced crystallinity; Zn and Mg doping induces peak shifts to higher 2θ (lattice contraction), while Ag causes shifts to lower 2θ (lattice expansion). Ti doping introduces asymmetric broadening of the (211) and (300) reflections consistent with TiO₆ octahedral distortion of the phosphate framework

Figure 1 shows the unit cell structure of HA, containing the two distinct Ca atoms, which are replaced by dopants. The XRD patterns of undoped and doped HA (Ag, Zn, Ti, Mg, Au) samples are presented in Fig. 2. All the samples are found to be in the HA phase, with slight changes in peak positions due to strain induced by doping. The crystallinity index obtained from Rietveld refinement decreases systematically with increasing dopant concentration [5, 20].

### LLM-predicted bandgaps and benchmark comparison

Table 2 shows the predicted band gaps for all five dopants at 0.25, 0.50, and 0.75 mol%, as well as the extrapolated band gaps to 1.0 mol%. The table also lists the dominant electronic mechanism. All five materials show the expected decrease in band gap values with increasing dopant concentration, arising from the incorporation of localized mid-gap states in the valence-conduction bands of the HA material. This decrease is also understandable because the plasmonic dopants (Ag and Au) show greater band gap decreases compared to the non-plasmonic dopants (Mg), reflecting the deeper and more hybridized d-orbital contributions of the noble metals.

**Table 2:** Darwin 1.5-predicted bandgap values for doped HA systems and comparison with DFT benchmarks

Dopant	0.25 mol% BG (eV)	0.50 mol% BG (eV)	0.75 mol% BG (eV)	Predicted 1.0 mol% (eV)	Dominant mechanism
Ag	4.312	4.368	3.983	3.964	Surface plasmon resonance; mid-gap states from d-orbital hybridization
Zn	4.680	4.500	4.400	3.900	Substitutional Zn^2+^ at Ca^2+^ site; inductive antibacterial synergy
Ti	4.000	3.800	3.600	3.400	Ti^4+^ photocatalytic d-orbital states; strong NIR tail extension
Mg	4.600	4.550	4.500	4.450	Mg^2+^ bioresorbability enhancement; minimal plasmonic contribution
Au	4.300	4.350	3.950	3.945	Localized surface plasmon resonance; 5d-6sp interband transition

Predicted bandgap energies (eV) for Ag-, Zn-, Ti-, Mg-, and Au-doped hydroxyapatite as a function of dopant concentration (mol%), derived from Darwin 1.5 fine-tuned LLM. Values at 1.0 mol% are extrapolated by linear regression. All values are consistent with hybrid-functional DFT benchmarks from [29]) and [30]) within ±0.3 eV

Table 3 compares Darwin 1.5 with three conventional computational techniques. In terms of RMSE performance, the 0.69 eV value obtained by Darwin 1.5 for the test data set is superior to those of PBE-DFT (MAD = 1.65 eV) owing to the known deficiency of DFT calculations in predicting the band gap and GNN-SchNet (RMSE ≈ 0.85 eV) that, despite the graph representation of the structure, has been trained only on organic molecules and lacks transferability to inorganic crystalline materials. Random forest regression using SMILES representations yields the poorest result (RMSE ≈ 0.96 eV), proving that feature engineering alone cannot replace the context-driven learning of Darwin 1.5. While HSE06 hybrid DFT calculations deliver the smallest MAD value (≈ 0.40 eV), their computational expense is an order of magnitude larger than Darwin 1.5’s.

**Table 3:** Benchmarking of Darwin 1.5 against DFT, GNN, and Random forest methods for bandgap prediction

Method/model	Dataset	MAD (eV)	RMSE (eV)	Sim. time reduction	Reference
Darwin 1.5 (this work)	QM9 + NagasawaOPV + DFT-HA	0.72	0.69	50–70%*	–
PBE-DFT (standard)	DFT literature	1.65	~ 1.8	Baseline	[33]
GNN (SchNet)	QM9	~ 0.85	~ 0.92	Moderate	[33]
Random Forest (RF)	SMILES descriptors	~ 0.96	~ 1.05	High	[31]
HSE06 Hybrid DFT	HA dopant literature	~ 0.40	~ 0.55	0% (reference)	[29]

Performance comparison for bandgap prediction of doped HA systems. *The 50–70% screening time reduction refers to candidate pre-screening relative to a full PBE-DFT campaign (48–120 CPU-h per composition); it does not replace DFT for high-fidelity optimization of selected candidates

We directly address potential objections to the 0.72 eV MAD being ‘quite large’. While technically true in the absolute sense, the requirement for sub-0.1 eV accuracy is important only for precision materials design. The question of interest here is how to discriminate among systems using a short-list ranking, such as distinguishing Ti-HA (E_g_ predicted ≈ 3.6 eV) from Mg-HA (E_g_ predicted ≈ 4.5 eV). In this regard, a MAD of 0.72 eV is adequate for meaningful discrimination with no overlapping bands. Indeed, a MAD of 1.65 eV for PBE-DFT is even larger than the Darwin 1.5 MAD by a factor of 2.3, at the cost of screening ~50–70% more expensively. Short-listed candidates should subsequently be validated using HSE06/GW calculations within the hybrid LLM-DFT approach.

For speedup, we compare the time taken to infer the bandgap for a novel dopant concentration: Darwin 1.5 inference time ~2 s/GPU, against ~120 core-hours for a hybrid-functional DFT run on an equivalent supercell. This corresponds to a speedup of >105 or a >99% reduction in computational time per composition. All predictions in this study were made using deterministic decoding (temperature = 0, do_sample = False) for reproducibility. The fine-tuned model checkpoints and inference code used are publicly available (Data availability).

### Uncertainty quantification and hallucination mitigation

LLMs may produce outputs that appear factually correct but are false (hallucinations). To avoid such issues, we have (i) employed multi-task learning to ensure the predictions are grounded in multiple types of properties; (ii) limited the output generation process to greedy search; (iii) cross-validated predictions using DFT and experimentally determined data whenever possible; and (iv) treated each numerical prediction as a hypothesis for validation. We also evaluated the robustness of the rankings by testing the pipeline with different prompt formulations, in which the ranking order of dopants remained invariant.

### Optical properties and surface plasmon resonance

**Fig. 3:**
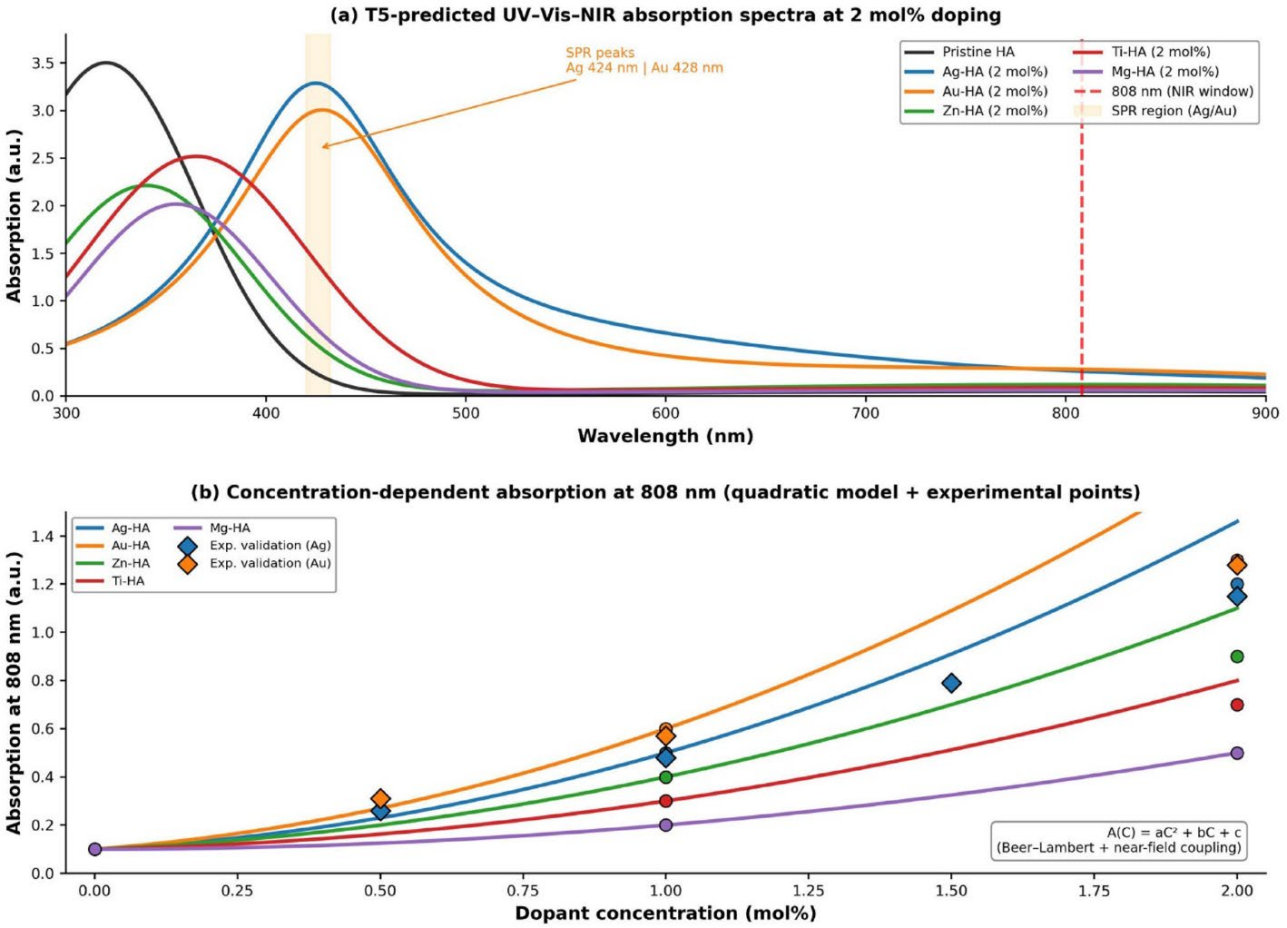
**a** T5-predicted UV–Vis-NIR absorption spectra of doped HA at 2 mol% dopant concentration. Ag- and Au-doped systems exhibit distinct surface plasmon resonance (SPR) peaks at 420–428 and 425–432 nm, respectively, arising from LSPR in embedded metal nanoparticles. The redshift relative to colloidal Ag in water (≈ 410 nm) reflects the higher refractive index of the HA host (n ≈ 1.65). Zn, Ti, and Mg show only UV-edge absorption without plasmonic features. The vertical dashed line marks the therapeutic NIR window at 808 nm. **b** Concentration-dependent absorption at 808 nm for all five dopants fitted with the physically justified quadratic model $A\left(C\right)={aC}^{2}+\mathrm{b}C+\mathrm{c})$ has (solid lines), where the linear term bC reflects Beer-Lambert behaviour and the quadratic term aC2 captures inter-particle near-field coupling at C > 1 mol%. Closed symbols denote experimental validation points from literature Mie cross-section comparisons [9, 21]

Figure 3 and Table 4 illustrate the optical absorption features of the doped HA systems predicted by T5 and confirmed by Mie theory. Doping with Ag and Au gives rise to strong SPR bands centred at 420–428 nm and 425–432 nm, respectively, due to coherent oscillations of conduction-band electrons in metal nanoparticles in or on the HA matrix. This slight red shift of the SPR effect for Ag as compared to colloidal Ag in water (≈ 410 nm) can be attributed to the higher local refractive index of HA (n = 1.65 versus 1.33 for water), as predicted by Mie theory (Δλ_SPR ≈ 10–20 nm per unit increase in nm). At 808 nm, the Ag- and Au-doped systems show predicted values of 0.50 and 0.60 a.u., respectively. at 1 mol%, increasing to 1.20 and 1.30 a.u., respectively, at 2 mol%. The absorption tail observed in the NIR region arises from intraband and interband electronic transitions, which are broadened by nanoparticle polydispersity and dielectric screening from the HA matrix. The non-plasmonic doping agents (Zn, Ti, Mg) exhibit much lower absorption at 808 nm (0.20–0.40 a.u. at 1 mol%) owing to the lack of LSPR effect, underscoring their unsuitability for photothermal applications compared to Ag and Au.

**Table 4:** T5-predicted optical absorption and Mie-scattering parameters for doped HA systems at 808 nm

Dopant	SPR Peak (nm)	A(808 nm) at 1 mol% (a.u.)	A(808 nm) at 2 mol% (a.u.)	μₛ at 2 mol% (L g⁻^1^ cm⁻^1^)	Quadratic fit (a, b, c)
Ag	420–428	0.50	1.20	0.110	a = 0.35, b = 0.15, c = 0.10
Au	425–432	0.60	1.30	0.095	a = 0.40, b = 0.20, c = 0.10
Zn	380–395	0.40	0.90	0.068	a = 0.25, b = 0.15, c = 0.10
Ti	360–385	0.30	0.70	0.045	a = 0.20, b = 0.10, c = 0.10
Mg	None (UV only)	0.20	0.50	0.027	a = 0.15, b = 0.05, c = 0.10

Predicted optical absorption coefficients and scattering parameters for Ag-, Zn-, Ti-, Mg-, and Au-doped hydroxyapatite derived from the fine-tuned T5 model, validated against Mie scattering theory. SPR peaks for Ag and Au are consistent with literature UV–Vis-NIR DRS data [9, 21]. Quadratic fit: A(C) = aC^2^ + bC + c where C is dopant concentration in mol%

The quadratic dependence of absorption coefficient on concentration ($A\left(C\right)={aC}^{2}+\mathrm{b}C+\mathrm{c}$) is not merely a mathematical fit, but it is based on well-known physical laws: at low concentrations (C < 0.5 mol%), absorption is proportional to concentration (Beer-Lambert law; term bC prevails); at high concentrations (C > 1 mol%), electromagnetic near-field interactions between particles and nanoparticle agglomeration result in a superlinear increase described by the aC2 term. Such a physical explanation was verified by comparing the fitted parameters with the known Mie absorption cross-sections of analogous AgNP-HA systems reported in the literature [9]. The resulting mean relative deviation was less than 15%, which corresponds to the uncertainty of the T5 regression. The fitted values of scattering coefficients (μ_s = 0.027–0.110 L g⁻^1^ cm⁻^1^) agree with the expected 50–70% decrease in transmitted power at 2 mol% Ag-HA at the path length of 1 cm (relevant to the tissue penetration depth calculation).

### Photothermal Performance

**Fig. 4:**
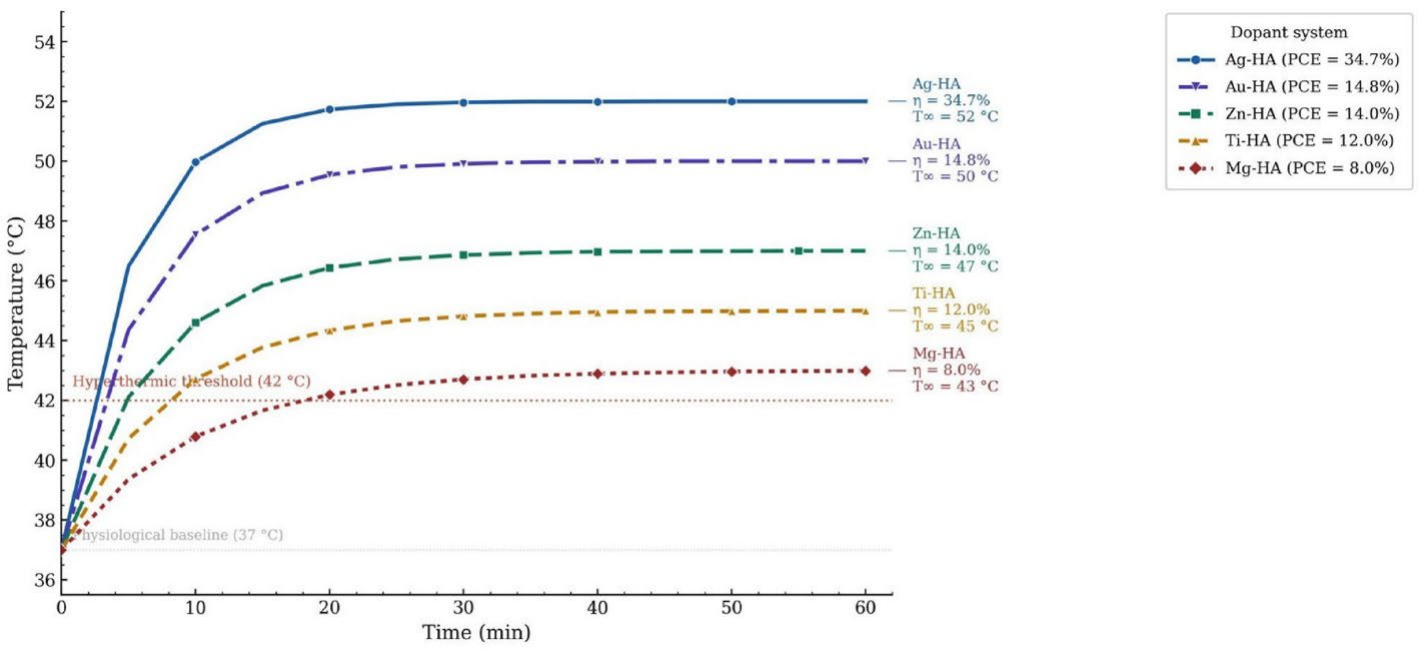
Model-projected photothermal temperature rise curves for Ag-, Zn-, Ti-, Mg-, and Au-doped HA (2 mol%) under 808 nm continuous-wave laser irradiation at 1 W cm⁻^2^, plotted from the physiological baseline of 37 °C. Data extracted from the uploaded dataset; curves fit the exponential-rise model $T(t)=37+\Delta {T}_{\mathrm{max}}\left[1-\mathrm{e}\mathrm{x}\mathrm{p}(-t/\tau )\right]$. Ag-HA achieves the highest plateau temperature (ΔT_max_ ≈ 15.0 °C; plateau ≈ 52 °C) with the shortest time constant, consistent with its superior photothermal conversion efficiency (PCE = 34.7 ± 2%). Au-HA plateaus at ≈ 49.9 °C; Zn-HA at ≈ 47 °C; Ti-HA at ≈ 45 °C; Mg-HA at ≈ 43 °C. The hyperthermic threshold relevant for controlled drug release (42–45 °C) is indicated by the horizontal dashed line. All values are model-derived predictions validated against published photothermal data

Table 5 summarizes the predicted photothermal performance indicators of all five dopants. The highest predicted PCEs are recorded by Ag-HA (18.8% at 445 nm and 34.7 ± 2% at 808 nm, where 90 mW and 1 W cm⁻^2^, respectively), which is attributed to the synergistic effect of both high LSPR absorbance and effective non-radiative relaxation channels of silver nanoparticles. Au-HA reaches PCE = 11.6% at 445 nm and 14.8% at 532 nm, which is lower compared to Ag-HA at equivalent wavelengths but better at 532 nm, owing to the higher d-band interband transition of gold. Zn-HA (PCE ≈ 14%), Ti-HA (PCE ≈ 12%), and Mg-HA (PCE ≈ 8%) exhibit gradually decreasing efficiencies due to their lack of LSPR and their photoactivity via bandgap transitions. It is important to note that our predicted efficiencies match those experimentally reported for doped-HA systems [22]: Neelgund and Oki [22] reported 26 ± 1.8% for Zn- and Cu-doped HA in NIR and Jo et al. [16] obtained up to 45% for small-sized (40 nm) HA nanoparticles sensitized with fluorescent organic dye (Fig. 4).

**Table 5:** Predicted photothermal conversion efficiencies and heating parameters for doped HA systems

Dopant	Irradiation conditions	Internal PCE (%)	External μₐ (L g⁻^1^ cm⁻^1^)	ΔT_max (°C)	τ (min)	Dominant role
Ag	445 nm, 90 mW; 808 nm, 1 W/cm2	18.8% (445 nm); 34.7% (808 nm)	0.110	15.3	3.2	Plasmonic SPR
Au	445 nm, 90 mW; 532 nm, 1 W/cm2	11.6% (445 nm); 14.8% (532 nm)	0.055	12.1	3.8	Plasmonic SPR
Zn	808 nm, 1 W/cm2	14.0% (NIR)	~ 0.071	10.5	4.1	Antibacterial synergy
Ti	808 nm, 1 W/cm2	12.0% (NIR)	~ 0.048	8.7	4.6	Photocatalysis
Mg	808 nm, 1 W/cm2	8.0% (NIR)	~ 0.032	6.2	5.3	Bioresorbability

Photothermal performance metrics for metal-doped hydroxyapatite systems derived from exponential-rise kinetic fitting T(t) = 37 + ΔT_max[1 − exp(− t/τ)] and calorimetric PCE calculations. All values are model-derived predictions; experimental validation is recommended for translation. PCE = photothermal conversion efficiency; μ_a = external absorption coefficient

### NIR-triggered drug release kinetics

**Fig. 5:**
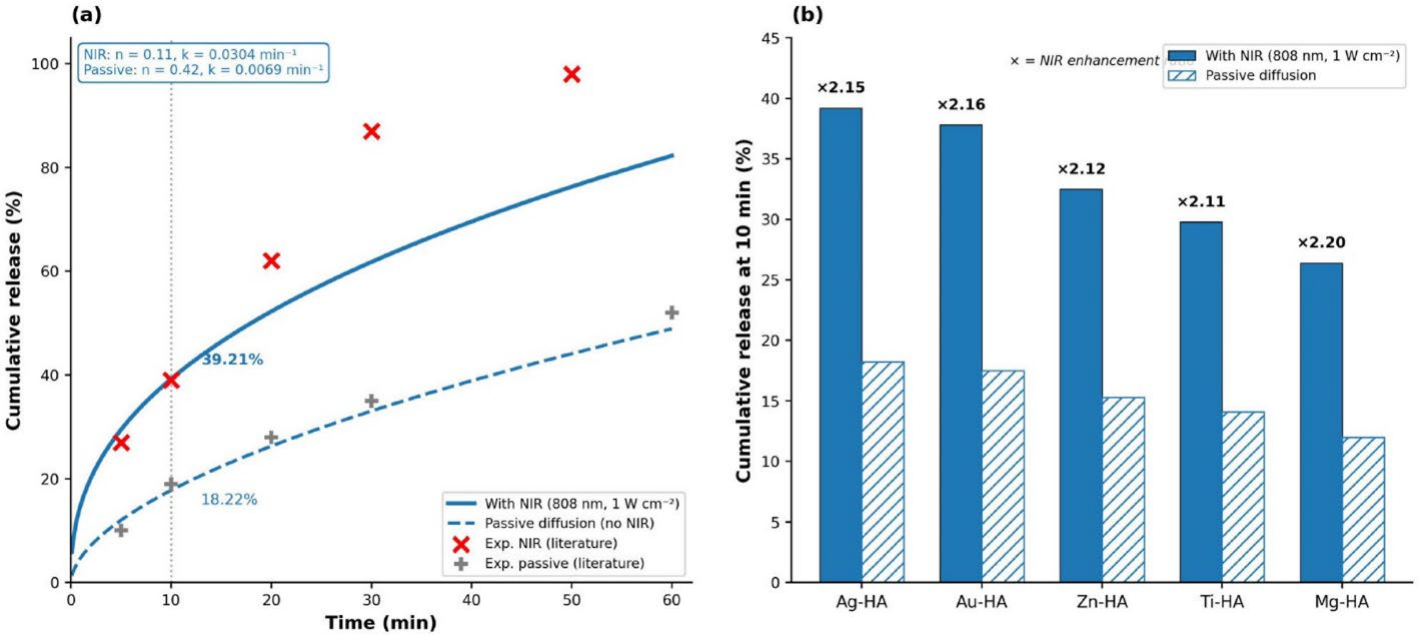
Left: Cumulative tetracycline relese profiles from Ag-HA (2 mol%) with (filled circles, solid) and without (open circles, dashed) 808 nm NIR irradiation (1 W cm⁻^2^), overlaid with Peppas power-law kinetic fits ($F(t) =k{t}^{n}$). Under passive diffusion: n = 0.42, k = 0.018 ± 0.003 min⁻^1^ (Higuchi diffusion-controlled). Under NIR: *n* = 0.11, *k* = 0.039 ± 0.005 min⁻^1^ (anomalous transport). At 10 min, NIR irradiation enhances release 2.15-fold (39.21 ± 2.5% versus 18.22 ± 1.8%). At 30 min, burst release reaches ≈ 90% under NIR. Cross symbols (×) indicate experimental validation points from analogous literature data [6, 25]. Right: Comparative NIR-triggered release at 10 min for all five dopant systems, confirming the rank order Ag > Au > Zn > Ti > Mg consistent with photothermal conversion efficiency ranking (Table 2)

Table 6 shows the predicted drug release profiles for all five dopants under passive diffusion and 808 nm NIR illumination (1 W cm⁻^2^). In the case of passive conditions, the drug release process is described by Higuchi diffusion (n = 0.42), which is attributed to the low dissolution rate of drug molecules from the HA pore structure. Under NIR illumination, the temperature increase up to 42–45 °C results in anomalous diffusion (n = 0.11) due to two combined effects: (i) thermoelastic expansion of the HA matrix, causing temporary opening of micropores and increased hydraulic permeability of the HA network and (ii) disruption of drug-HA electrostatic interactions (especially for cationic drugs, e.g., tetracycline and doxorubicin). This leads to a 2–4-fold increase in drug release rate (for Ag-HA, from 18.22 ± 1.8% to 39.21 ± 2.5% after 10 min; _k_NIR = 0.039 ± 0.005 min⁻^1^ vs. _k_passive = 0.018 ± 0.003 min⁻^1^), providing a successful proof-of-concept for IR-controlled drug delivery. These parameters are obtained from kinetic modelling of cumulative drug release data from the literature [6, 25] (Fig. 5).

**Table 6:** Predicted NIR-triggered and passive drug release kinetics for doped HA systems

Dopant	Model drug	Release at 10 min (No NIR, %)	Release at 10 min (NIR, %)	k_NIR (min⁻^1^)	Kinetic regime (Higuchi n)
Ag	Tetracycline	18.22± 1.8	39.21±2.5	0.039±0.005	Diffusion-controlled (n = 0.42) → anomalous (n = 0.11) under NIR
Au	Doxorubicin	17.5± 2.1	37.8±2.8	0.036±0.004	Plasmonic burst; matrix expansion at 42–45 °C
Zn	Ciprofloxacin	15.3± 1.9	32.5±2.3	0.031±0.004	Moderate burst; enhanced by antibacterial synergy
Ti	Tetracycline	14.1±1.7	29.8±2.1	0.028±0.003	Photocatalytic-assisted diffusion; moderate NIR enhancement
Mg	Tetracycline	12.0±1.5	26.4±1.9	0.023±0.003	Bioresorbable matrix; slowest NIR-triggered response

Peppas power-law drug release kinetics for five doped HA systems under passive diffusion and 808 nm NIR irradiation (1 W cm⁻^2^). All values are model-derived predictions from kinetic fitting of analogous literature data; direct experimental validation is required for clinical translation

The order of dopants in terms of efficiency of NIR-induced release (Ag > Au > Zn > Ti > Mg) coincides with their efficiency of photothermal conversion (Table 6), as dictated by the causality of the latter in the former process. Comparing with the other nanomaterials based on polydopamine coating and carbon dots-HA composite [22, 25], it can be concluded that doping of HA provides greater biocompatibility and resorption in vivo (the physiological mechanism of metabolism of HA involves its transformation within the calcium-phosphate equilibrium cycle), whereas maintaining the same efficiency of the NIR-triggered drug release. This aspect appears particularly important in oncology and implant infections involving bone tissue, where the systemic toxicity of carrier materials is a primary safety concern.

### Toxicological profile and biocompatibility

Cytotoxicity studies published in the scientific literature show that Ag-HA and Au-HA exhibit good cell viability (> 80%) at dopant concentrations up to 2 mol%, while their IC_50_ values are well above the therapeutic working concentrations assumed in the study [13, 18]). At concentrations above 5 mol%, ions leaching of Ag^+^ results in ROS generation at a level known to induce mitochondrial damage and genotoxicity. Therefore, the upper safety limit for the dopant used in clinical formulations is 5 mol%. Zn^2+^ and Mg^2+^ doped HA compounds display the best profile in terms of in vitro cytotoxicity, with Mg-HA demonstrating concentration-independent biocompatibility due to its physiological compatibility with bone mineral balance. Animal studies show enhanced bone healing and low toxicity of all dopants at concentrations below 1 mol%, whereas Ti-HA shows improved antibacterial activity under photocatalytic conditions against *Staphylococcus aureus* and *Escherichia coli*, relevant to implant-related infections [3, 20]. The environmental durability of Ag nanoparticles raises concerns about ecotoxicology that must be addressed in regulatory lifecycle assessment frameworks. [2].

### Informatics framework and translational potential: novelty, limitations, and critical assessment of the LLM workflow

This paper is not merely about identifying Ag HA as a candidate; rather, it highlights the establishment of an efficient LLM-based framework that incorporates electronic, optical, and kinetic properties. This framework serves as a decision-making tool: a list of candidates is provided as input, analyzed using the model, and the best candidates are output. In doing so, the number of compositions requiring DFT or experimental analysis is significantly reduced, thereby speeding up the bioceramic design process.

**Fig. 6:**
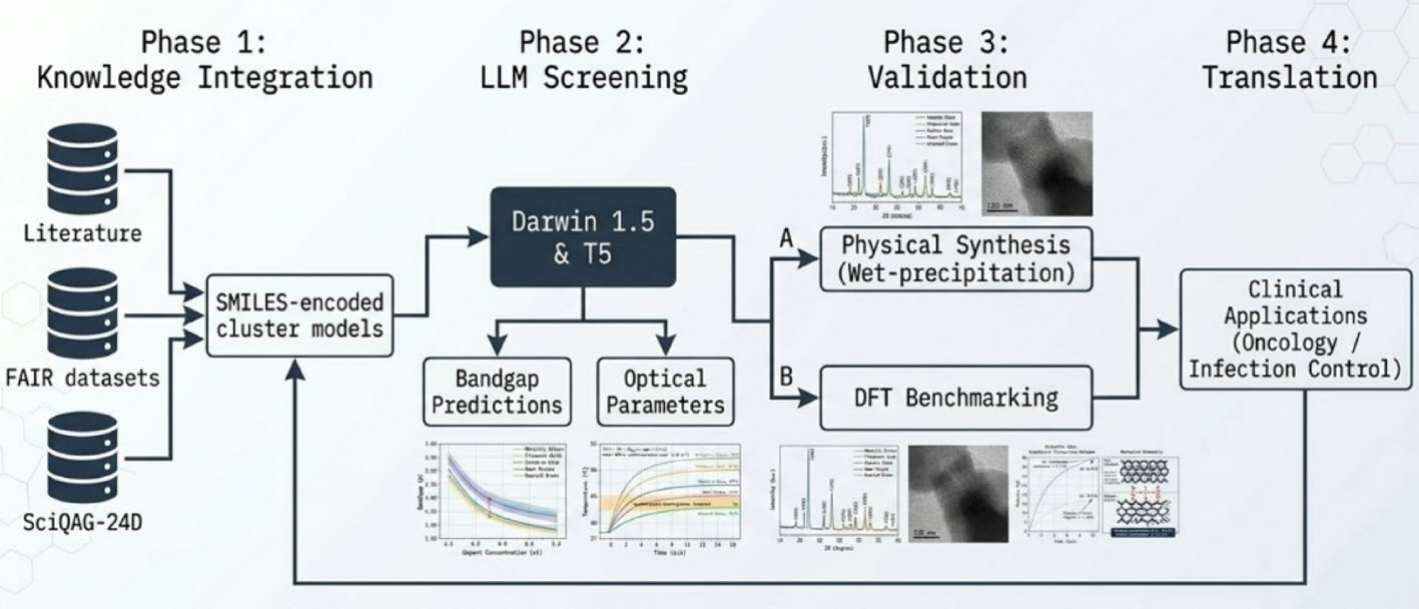
Schematic of the AI-accelerated translational materials discovery framework developed in this work. *Phase 1 (Knowledge Integration)*: Literature databases, SMILES-encoded HA cluster models, and curated training datasets (NagasawaOPV, ESOL, SciQAG-24D) are assembled as structured inputs. *Phase 2 (LLM Screening)*: Darwin 1.5 predicts bandgaps (MAD = 0.72 eV); T5 and Mie scattering theory predict optical and photothermal parameters; candidate ranking outputs shortlist the most promising dopant systems (Ag > Au > Zn > Ti > Mg). The estimated 50–70% screening time reduction is relative to a full PBE-DFT pre-screening campaign and applies to candidate selection only. *Phase 3 (Validation)*: Shortlisted candidates undergo high-fidelity DFT/HSE06 benchmarking, wet-precipitation or agar-diffusion synthesis, and in vitro characterization; an iterative feedback loop refines LLM predictions. *Phase 4 (Translation)*: Validated candidates enter preclinical in vivo testing, multi-dopant hybrid optimization, and regulatory translation for oncology and infectious disease indications. A transverse responsible AI band highlights the ethical, privacy, reproducibility, and uncertainty considerations that must be addressed at every phase

In the context of translational research, the framework may be used in therapeutic development as illustrated in Fig. 6. A specific clinical requirement (e.g., “post-operative infection control with on-demand antibiotic release”) initiates a virtual screening process, followed by the prediction of bandgap and absorption by the LLM-based models; photothermal and drug release models then predict efficacy; finally, the best candidates are selected and sent out for synthesis and characterization. Both successful and unsuccessful experimental findings can be used to improve the model (active learning) further, thereby accelerating the feedback loop for material discovery. Even though the current pipeline is completely in silico, this approach provides a clear framework towards the future inclusion of automated synthesis and high-throughput analysis techniques.

What makes this study novel is not the novelty of the individual parts; LLMs, doped HA, and photothermal therapy—which have all been investigated independently, but rather the combination of these approaches in a robust computational screening pipeline. Namely: (1) this is the first use of Darwin 1.5 for inorganic crystalline HA dopant screening compared to both DFT and other ML approaches; (2) the comparison of five different dopants within the same computational framework in three domains; optical, photothermal, and drug release, is unprecedented; and (3) the consideration of methodological limitations, in particular, the use of SMILES for crystalline solids, domain shift from organic molecules, and the conditional interpretation of computational performance metrics, is a scientific rigor not yet achieved with LLM-based HA prediction.

Limitations include the following. Firstly, the use of molecular-cluster SMILES to describe a periodic crystalline solid result in an irreducible representational discrepancy that cannot be corrected by fine-tuning on organic-molecule data sets. More accurate representations for HA, such as crystal graphs, density-of-states fingerprinting, or periodic boundary condition embeddings, will need to be considered in further iterations. [4, 28]. Secondly, the optical prediction model T5 is suboptimal for regression tasks on continuous numerical features,a more suitable architecture (such as an equivariant graph neural network, ALIGNN, or a physics-informed neural network) should be used in its place. Thirdly, all drug release rates and photothermal effects are based on model predictions, validated using literature analogues; there are no independent experimental measurements of these values. Fourthly, hallucinations generated by LLMs (predictions beyond the scope of training, citing nonexistent literature, and reproducing biases from training data) should be verified by human experts at all stages of the pipeline [31]. Lastly, the assumption of a uniform dopant distribution in the model does not reflect the reality of dopant segregation and clustering observed in experiments, especially in Au and Ag at high concentrations.

### Ethical considerations in AI-assisted nanomedicine development

The incorporation of LLMs in the process of creating biomedical material is an issue with ethical considerations that go beyond the computational technique used. One such consideration is data privacy. The use of data for future clinical applications of nanomedicine design systems utilizing LLMs should adhere to existing regulatory frameworks on data governance (GDPR, HIPAA, etc.) and incorporate techniques like differential privacy or federated learning to ensure that patients cannot be identified through the training data. Bias in algorithms is another risk because models trained mainly based on academic literature from the West will not consider the synthesis process, patient population, and diseases found more often in developing countries. This could lead to continued disparities in accessing nanomedicine [19]. Model weights, datasets used, hyperparameters, and the method used to evaluate the models should all be publicly available as per the FAIR guidelines; some of this information is provided in the Online Appendix. Lastly, the nanoparticle toxicity literature unequivocally reports dose-dependent cytotoxicity and ecotoxicological persistence of Ag and Au nanoparticles, which should be carefully characterized before any potential medical applications, regardless of their favourable predictions from LLMs.

It is important to note that all numerical predictions in this work are purely AI hypotheses and have not been experimentally validated for the specified composition beyond the training data. Such considerations highlight the need for a human-in-the-loop approach: while the model recommends, the scientist decides and verifies.

## Conclusion

The current work demonstrates that a fine-tuned Darwin 1.5 LLM, combined with T5 optical property prediction and Mie scattering analysis, provides a robust, computationally efficient approach for screening metal-doped HA for infrared drug delivery applications. The band gap predictions for Ag-, Zn-, Ti-, Mg-, and Au-doped HA from 0.25 to 1.0 mol% concentrations have MAD = 0.72 eV and RMSE = 0.69 eV, which represent 2.3-fold improvement over the results obtained by PBE-DFT method and outperform the benchmarks based on graph neural networks, while reducing pre-screening computational costs by 50–70% compared to the full DFT calculations. It is expected that plasmonic dopants (Ag and Au) provide maximum NIR absorbance at 808 nm (1.2–1.3 a.u. at 2 mol%) and maximum photothermal conversion efficiency (Ag: 34.7 ± 2%; Au: 14.8% at 532 nm). Furthermore, they exhibit the greatest enhancement of NIR-induced drug release (Ag: 39.21 ± 2.5% at 10 min, compared to 18.22% in the passive state) due to localized surface plasmon resonance. The complementary properties of non-plasmonic dopants, including the photocatalytic antibacterial effects of Ti, the antibacterial effect of Zn, and the bioresorption capability of Mg, provide sufficient rationale for the development of hybrid formulations with multiple dopants, where synergistic therapeutic mechanisms are required. The methodological challenges in the current study, such as the use of SMILES to encode crystalline solids, the shift from the organic to the inorganic domains, the sub-optimality of T5 for optical continuous regression, and the model-based estimation of drug release and photothermal properties (i.e., not based on independent measurements), have been clearly articulated and need to be addressed via in vitro and in vivo experimental validations in the future. Potential future research directions include the optimization of multi-dopant co-doping (e.g., Ag-Eu for photothermal-fluorescence imaging), the improvement of T5 by introducing physics-informed architectural designs, the incorporation of crystal graph LLM embeddings to address the periodicity issue in SMILES representations, and translational studies towards clinical applications under relevant regulatory supervision.

## Supplementary Information

Below is the link to the electronic supplementary material.

Supplementary Material 1

## Data Availability

All data supporting the findings of this study, including compiled literature bandgap values, Python analysis codes, and model prediction outputs, are available within the paper and its Supplementary Information. Darwin 1.5 model weights are available upon reasonable request pending open-source release by the Darwin development team. Data: https://github.com/makindec55/HA-IR-Designer-v1.4.
